# A Hidden Checkpoint: Bidirectional pH Remodeling of the Immunological Synapse in Cancer, Methods, and Therapeutic Opportunities

**DOI:** 10.1155/jimr/5768926

**Published:** 2026-04-29

**Authors:** Aurélie Guillemette, Eva Gez, Sofia Titah, Yasmine Touil

**Affiliations:** ^1^ PERSTIM Lab, CRCLille, Inserm U1366, CNRS UMR9020, ONCOLille, University of Lille, Institut Pasteur Lille (IPL), CHU Lille, F-59000, Lille, France, chru-lille.fr

**Keywords:** acidic pH, calcium signaling, immune synapse, immunotherapy, methodological approaches

## Abstract

The immunological synapse (IS) is a nanoscale platform that coordinates T cell activation, cytoskeletal polarization, Ca^2+^ signaling, and the directed secretion of lytic granules. In cancers, an acidic tumor microenvironment (TME; extracellular pH_e_ ~ 6.4–6.8) imposes a biophysical and metabolic stress that could destabilize this interface. Experimental studies indicate that even modest acidification could significantly reduce integrin‐dependent adhesion strength, delay actin clearance at the synapse, and suppress store‐operated Ca^2+^ entry, thereby leading to marked decreases in cytokine production and cytotoxic granule release. In parallel, tumor cells often maintain relative intracellular alkalinity by enhancing proton export via transporters such as NHE1, MCT1/4, and CAIX, thereby reinforcing cortical actin “shielding,” metabolic resilience, and resistance to perforin‐mediated killing. These asymmetric pH adaptations may therefore establish a hidden checkpoint at the IS that favors tumor survival. We synthesize current evidence on pH‐dependent regulation of actin dynamics, integrin activation, mitochondrial function, and Ca^2+^ channels (Orai1/STIM1); highlight key methodological gaps, including the lack of approaches combining real‐time intra‐ and extracellular pH and Ca^2+^ imaging; and discuss enabling technologies such as microfluidic platforms, genetically encoded pH sensors, and multiparametric single‐cell assays. Finally, we outline therapeutic strategies aimed at modulating pH (buffers, inhibitors of NHE1, MCTs, V‐ATPases, or CAIX) or engineering pH‐resistant effector cells and consider how these approaches could synergize with immune checkpoint blockade, CAR‐T cells, and bispecific antibodies. Viewing acidosis as a druggable checkpoint reframes the IS as a bidirectional, pH‐tuned system and suggests testable paths to restore antitumor immunity.

## 1. Introduction

The immunological synapse (IS) is a dynamic and highly structured nanoscale interface that orchestrates T cell activation, cytotoxicity, and long‐term fate decisions. Efficient IS formation ensures proper polarization of the cytoskeleton, calcium signaling, and directed secretion of effector molecules, which together determine the success of T cell–mediated tumor cell killing.

Within the tumor microenvironment (TME), however, immune surveillance is challenged by a range of immunosuppressive factors. Among these, extracellular acidity (pH ~ 6.4–6.8) has emerged as a key but underappreciated regulator of immune cell function [1, 2]. Tumor‐driven acidosis results from altered metabolism, including aerobic glycolysis (the Warburg effect) and poor vascularization, creating a hostile biochemical environment for infiltrating lymphocytes [1, 3].

We hypothesize that acidic pH acts as a hidden immune checkpoint, directly impairing the structural and signaling integrity of the IS [4]. Specifically, acidosis may destabilize cytoskeletal remodeling, perturb calcium influx, and alter bidirectional crosstalk between T cells and tumor cells, ultimately promoting immune escape [5, 6].

This review will examine (i) the mechanical and molecular consequences of extracellular acidosis on IS stability, cytoskeleton, and Ca^2+^ dynamics; (ii) intracellular pH regulation and its role in T cell and tumor cell adaptation; (iii) the bidirectional effects of acidity on both T cells and cancer cells; (iv) methodological approaches to monitor intra‐ and extracellular pH in the context of IS; and (v) emerging therapeutic strategies to neutralize acidity and restore immune synapse function.

## 2. The IS and the Potential Impact of Extracellular and Intracellular Acidity

### 2.1. The IS: Structure and Dynamics

The IS is a highly organized supramolecular structure that forms at the interface between T cells and antigen‐presenting cells (APCs) or tumor cells. It coordinates antigen recognition, intracellular signaling, cytoskeletal remodeling, and polarized secretion of effector molecules [7]. Structurally, the IS is composed of concentric supramolecular activation clusters (SMACs): a central SMAC enriched in T cell receptor (TCR)–MHC interactions, a peripheral SMAC (pSMAC) dominated by integrins (LFA‐1/ICAM‐1), and a distal SMAC containing CD45 and other molecules involved in regulation. Together, these domains orchestrate the spatiotemporal dynamics of T cell activation and effector function [8]. IS formation is not static but a cyclic process of engagement and disengagement as T cells continuously scan for antigens within tissues [8]. Efficient IS assembly requires reorganization of the actin cytoskeleton and polarization of the microtubule‐organizing center (MTOC) toward the synaptic interface, which aligns secretory vesicles and mitochondria near the contact site [9]. Mitochondria play a particularly crucial role by buffering local Ca^2+^ and sustaining signaling cascades [10, 11].

### 2.2. Acidity in the TME

The TME is frequently acidic, with extracellular pH (pH_e_) ranging from 6.4 to 6.8, compared to the physiological 7.4. This acidosis results from a combination of the Warburg effect (aerobic glycolysis), poor vascular perfusion, and accumulation of lactate [1, 12]. Beyond being a metabolic byproduct, acidity actively shapes immune responses, contributing to an immunosuppressive niche that fosters tumor survival and immune evasion [1, 2].

Seminal work by Huber and colleagues conceptualized cancer acidity as an “ultimate frontier of immune escape” [1]. Recent studies support this view by demonstrating that acidic pH directly impairs CD8^+^ T cell proliferation, cytokine production, and cytotoxic activity [3]. Importantly, extracellular acidosis is now recognized as a regulator of the IS, influencing its stability and the downstream signaling pathways necessary for effective immune killing.

### 2.3. Intracellular pH Regulation in T and Cancer Cells

Intracellular pH (pHᵢ) is tightly regulated in T lymphocytes, typically maintained between 7.0 and 7.4 under physiological conditions. This balance relies on proton transporters such as the Na^+^/H^+^ exchanger (NHE1), the vacuolar‐type H^+^‐ATPase (V‐ATPase), and bicarbonate transporters, which collectively sustain enzymatic activity and signaling pathways [2, 12, 13]. Upon TCR engagement, T cells undergo rapid metabolic reprograming that involves both glycolysis and oxidative phosphorylation (OXPHOS) [2]. These processes generate lactate and protons, transiently lowering pHᵢ. To preserve activation, T cells upregulate NHE1 activity to extrude protons, leading to intracellular alkalinization, an early and essential event for proliferation, IL‐2 production, and effector function [3]. Disruption of this pHᵢ regulation compromises IS formation, calcium signaling, and downstream cytotoxicity [3, 14].

In cancer cells, pH regulation follows an inverted pattern compared to T lymphocytes. While tumor cells are often exposed to an acidic extracellular environment (pH_e_ 6.4–6.8), they actively maintain a relatively alkaline intracellular pH (pHᵢ 7.2–7.6). This is achieved by overexpression of proton transporters such as NHE1, monocarboxylate transporters (MCT1‐4), and carbonic anhydrase IX (CAIX), which export protons and lactate into the extracellular space [12, 15]. This intracellular alkalinization supports anabolic metabolism, cytoskeletal remodeling, and resistance to apoptosis [16]. Importantly, an alkaline pHᵢ enhances actin polymerization [16–18] and the formation of a dense cortical cytoskeleton, which has been shown to impede CTL‐mediated lysis at the IS [9]. Notably, extracellular acidosis has been shown in multiple tumor models to upregulate the expression of immune checkpoint molecules such as PD‐L1, thereby promoting immune evasion [19, 20]. Although direct evidence demonstrating that tumor‐intrinsic intracellular alkalinization per se induces checkpoint ligand expression is currently lacking, it is plausible that pHi remodeling contributes to or potentiates these immunosuppressive programs. Establishing whether cancer cell alkalinization directly regulates immune checkpoint expression, therefore, represents an important and testable hypothesis for future studies.

Taken together, intracellular pH regulation diverges fundamentally between T cells and tumor cells: T cells require transient alkalinization to support activation and effector functions, whereas tumor cells exploit persistent alkalinization to reinforce survival, motility, and resistance to immune attack. This dichotomy positions pHᵢ as a potential targetable vulnerability within the tumor‐immune synapse.

### 2.4. Extracellular and Intracellular pH as Regulators of Cytoskeletal Dynamics: Relevance for the IS

#### 2.4.1. Extracellular pH and Cytoskeletal Remodeling

The cytoskeleton is highly sensitive to changes in extracellular acidity, with multiple studies demonstrating that pH exerts a direct control on actin dynamics, adhesion, and motility. Early work showed that lowering extracellular pH decreases the basal motility of cultured mouse microglia by reorganizing actin into thick stress fibers, thereby impairing cell migration [21]. Similar effects were reported in tumor models, where acidic pH promoted lamellipodia and filopodia reorganization, consistent with increased cytoskeletal plasticity that enables cancer cells to migrate and invade [22]. Extracellular pH can be sensed at the plasma membrane by proton‐responsive receptors, including a subset of G‐protein‐coupled receptors (GPR4, GPR68/OGR1, and GPR65/TDAG8) and non‐GPCR ion channels such as TRPV1 and acid‐sensing ion channels (ASICs) [23]. These sensors convert acidic signals into intracellular Ca^2+^ or cAMP responses and thus provide a potential molecular interface through which extracellular acidity could influence signaling at the IS. However, their direct contribution to synapse formation and function remains incompletely defined and warrants further investigation.

Integrins represent another critical cytoskeletal interface sensitive to extracellular pH. Paradise et al. [24] demonstrated that extracellular acidosis promotes the activation of integrin αvβ3 by favoring conformational changes that enhance its affinity for ligands. Since integrin activation triggers downstream actin polymerization and adhesion complex stabilization, pH fluctuations can profoundly reshape cytoskeletal anchoring and, by extension, cell–cell interactions. In the TME, where extracellular pH can fall to 6.4–6.8, these effects may drive adhesion remodeling in both tumor cells and immune infiltrates.

#### 2.4.2. Intracellular pH and Actin Regulation

pH_i_ also directly modulates cytoskeletal organization [23]. Actin dynamics are sensitive to pHi through the regulation of key actin‐binding proteins, including ADF/cofilin and talin. Members of the ADF/cofilin family function as pH‐responsive modulators of actin filament turnover [17, 23], with alkaline pHi favoring their severing activity by promoting cofilin release from membrane phosphoinositides and enhancing ADF‐actin interactions. In parallel, talin, a critical linker between integrins and the actin cytoskeleton, exhibits pH‐dependent actin binding through its C‐terminal domain, such that elevated pHi weakens talin‐actin interactions and facilitates adhesion remodeling [23]. Although these mechanisms have been characterized primarily in migratory or adherent cells, they provide a plausible molecular basis by which local pHi changes could influence actin reorganization, integrin dynamics, and mechanical stability at the IS.

#### 2.4.3. Cancer Cell Adaptation and Cytoskeletal Plasticity Under Acidosis

The role of acidic pH in promoting tumor cell cytoskeletal adaptation is well established. Huber et al. [1] described cancer acidity as a frontier of immune evasion, noting that cytoskeletal remodeling underlies changes in adhesion, migration, and resistance to cytotoxic attack. Importantly, these modifications extend beyond migration: actin reorganization at the tumor side of the IS has been reported to hinder cytotoxic lymphocyte killing by creating a dense cortical barrier that prevents granule delivery [9, 25]. The link between extracellular acidosis and this tumor‐intrinsic cytoskeletal defense remains underexplored, but conceptually, low pH could enhance actin barrier reinforcement, thereby stabilizing resistance.

#### 2.4.4. What Is Missing: T Cell Cytoskeleton and Acidic pH

While the role of acidic pH in tumor cytoskeletal remodeling is increasingly recognized, its impact on T lymphocytes remains poorly defined. Cytoskeletal polarization is central to T cell activation: following TCR engagement, actin remodeling supports the formation of the pSMAC, while MTOC polarization directs lytic granule secretion toward the central SMAC [7, 8, 26]. Small GTPases (Rac1, Cdc42, and RhoA) and actin regulators (Arp2/3, WASp, and formins) coordinate these rearrangements, ensuring proper synapse assembly and stability [27]. Despite detailed mechanistic knowledge of actin control in T cells, almost no studies have examined how extracellular or intracellular acidosis interferes with these processes. Given that acidosis alters integrin activation in other cell types [24], it is plausible that the critical LFA‐1/ICAM‐1 axis at the IS could be weakened under low pH, destabilizing adhesion and reducing cytotoxicity. Similarly, pHi‐dependent regulation of ADF/cofilin and talin may alter actin turnover at the synapse, impacting sustained signaling and killing capacity. Yet, direct evidence for these hypotheses is missing.

In summary, extracellular and intracellular acidosis are well established as modulators of cytoskeletal architecture in tumor cells [23]. However, their impact on T cell cytoskeletal remodeling during IS formation is largely unknown. No systematic studies have addressed how acidic pH alters integrin‐dependent adhesion, actin turnover, or MTOC polarization in lymphocytes. This represents a major blind spot, given the centrality of cytoskeletal remodeling for T cell activation and cytotoxicity. Investigating this axis could reveal how acidic pH functions as a hidden immune checkpoint, destabilizing IS architecture and enabling tumor immune escape. Bridging this gap requires integrating live‐cell imaging, microfluidic modeling of the TME, and functional assays of T cell killing under defined pH conditions. Such studies would not only advance our mechanistic understanding but also highlight novel therapeutic strategies, including buffering, ion transporter inhibitors, and engineered T cells resistant to acidosis.

### 2.5. Calcium Signaling Under Acidic Conditions

Ca^2+^ influx through calcium release activated calcium (CRAC) channels is a cornerstone of T cell activation. Sustained Ca^2+^ oscillations drive NFAT nuclear translocation and promote transcription of effector cytokines such as IL‐2. Recent work suggests that endogenously produced protons continually modulate the membrane potential, release from Ca^2+^ stores, and intracellular Ca^2+^ buffering, impacting Ca^2+^ signaling [28]. Acidic pH attenuates store‐operated calcium entry (SOCE), thereby reducing TCR‐induced Ca^2+^ oscillations [29]. In CD8^+^ T cells, this would translate into defective release of granzyme and perforin, ultimately impairing effector functions [30]. Moreover, mitochondria, which normally polarize to the IS to buffer Ca^2+^ and sustain oscillatory signaling, are strongly affected by extracellular acidosis [5]. Recent work demonstrated that low pH impairs mitochondrial respiration and limits ATP availability, creating a vicious cycle: Reduced Ca^2+^ entry compromises mitochondrial recruitment. In contrast, mitochondrial dysfunction further destabilizes Ca^2+^ homeostasis at the IS [5].

pHi also critically shapes calcium signaling. Alkalinization enhances CRAC channel activity and SOCE, sustaining NFAT‐dependent transcription [29]. By contrast, intracellular acidification inhibits SOCE by altering the electrochemical driving force and directly modulating CRAC channel gating [29]. Acidic pHᵢ also impairs mitochondrial Ca^2+^ buffering, thereby destabilizing the tight functional coupling between mitochondria and CRAC channels [31, 32].

A mechanistic explanation for this sensitivity has been provided by structural studies identifying pH‐sensitive amino acid residues in Orai1/STIM1 proteins [33]. Tsujikawa et al. [33] demonstrated that both extracellular and intracellular acidic conditions alter Orai1 conformation and gating, leading to impaired Ca^2+^ influx. More recently, Audero et al. [34] emphasized that tumor acidosis acts as a metabolic immune checkpoint by directly targeting Ca^2+^ signaling hubs. Together, these findings highlight that pH not only modulates the biophysics of CRAC channels but also could integrate extracellular and intracellular cues to shape the functional capacity of T cells during immune synapse formation.

## 3. Extracellular vs. Intracellular pH: Bidirectional Effects on T Cells and Tumor Cells at the IS

### 3.1. The IS as a Bidirectional Structure

The IS is not merely a platform for TCR signaling but a highly coordinated nanoscale interface where both T cells and tumor cells actively remodel their cytoskeleton and membrane organization. Traditionally, research has emphasized T cell polarization, actin clearance, and calcium mobilization as prerequisites for effective cytotoxicity [35]. However, accumulating evidence indicates that tumor cells are not passive recipients. Instead, they actively reorganize their cortical actin and microtubules to resist granule delivery and perforin pore formation [36]. Tumor cells can accumulate actin at the contact site, creating a barrier to perforin pores and granzyme entry [37]. The proposed tumor‐side “actin shield” at the IS is supported by growing but still limited experimental evidence. Across several solid tumor types, activation of the RhoA‐ROCK pathway has been consistently implicated in cortical actomyosin reinforcement, increased membrane tension, and resistance to perforin‐mediated killing [27, 37]. ERM (ezrin/radixin/moesin) proteins further contribute by anchoring cortical actin to the plasma membrane and stabilizing the tumor cell cortex during immune engagement [38]. In parallel, independent studies have demonstrated that tumor cells can rapidly recruit the endosomal sorting complex required for transport (ESCRT) membrane repair machinery to seal perforin‐induced membrane lesions, thereby limiting cytotoxic damage [27]. In contrast, Rac1‐driven actin polymerization appears more context‐dependent and is less uniformly validated across tumor entities. Importantly, while these pathways demonstrate that tumor cells actively remodel their actin cytoskeleton in response to immune attack, the integration of these mechanisms into a unified, pH‐modulated “actin shield” operating specifically at the IS remains partly hypothetical. Direct visualization and quantitative assessment of such a barrier, particularly under acidic conditions, are still lacking, and further studies will be required to establish its functional relevance. On the other side, T cells require precise actin clearance at the c‐SMAC to facilitate granule delivery. Extracellular acidosis may therefore tip the balance in favor of the tumor by simultaneously strengthening the tumor actin barrier and weakening T cell polarization. This bidirectional effect remains untested but could represent a hidden immune checkpoint imposed by the acidic TME. Within this context, pH regulation emerges as a powerful, yet underexplored, determinant of this reciprocal crosstalk.

### 3.2. Extracellular Acidosis: Asymmetric Impact on T Cells vs. Tumor Cells

Acidic pHe (pH_e_ ~ 6.4–6.8) is a well‐established hallmark of TME and the bone marrow niche in multiple myeloma [39]. This acidity primarily results from high rates of tumor glycolysis and proton extrusion through MCT. Crucially, extracellular acidosis does not affect all cell types equally. In T cells, exposure to acidic pH_e_ reduces motility, impairs TCR signaling, suppresses cytokine secretion [40], and impairs cytokine signaling [41]. Specifically, acidosis diminishes IL‐2 production and dampens Ca^2+^ fluxes, leading to impaired cytotoxic activity. In contrast, tumor cells exploit acidosis through a phenomenon known as the “reverse pH gradient.” Despite the acidic extracellular milieu, they sustain an alkaline intracellular pH via proton pumps and exchangers [23]. This intracellular alkalinization confers several advantages: It promotes survival, enhances glycolytic metabolism, and reinforces cytoskeletal remodeling critical for invasion and immune resistance [23, 25, 42].

Taken together, extracellular acidosis creates a functional asymmetry at the IS: T cells are weakened by intracellular acidification, whereas tumor cells maintain metabolic fitness and structural resilience by preserving intracellular alkalinity.

### 3.3. Intracellular Acidosis in T Cells: Dysregulated Signaling, Exhaustion, and Distinct pH‐Sensitivity According to the T Cell Subset

Intracellular acidification in T cells perturbs proximal TCR signaling, reducing phosphorylation of key components and impairing NFAT activation and nuclear translocation [29]. This disruption also compromises cytoskeletal dynamics, as evidenced by diminished actin clearance at the c‐SMAC, which delays the trafficking of lytic granules toward target cells [17, 37]. Sustained acidosis accelerates T cell exhaustion, characterized by increased expression of inhibitory receptors such as PD‐1, TIM‐3, and LAG‐3 [2, 43].

Importantly, the impact of pHi varies markedly across T cell subsets, reflecting profound differences in their metabolic wiring and signaling dependencies. Effector CD8^+^ T cells, which rely heavily on aerobic glycolysis to sustain rapid ATP production and biosynthetic fluxes, are susceptible to intracellular acidosis. Experimental studies have shown that modest decreases in pHi (0.2–0.4 units) are sufficient to dampen glycolytic enzyme activity, reduce Ca^2+^ influx, and impair actin remodeling, resulting in defective cytokine secretion and diminished cytotoxic granule exocytosis [5, 44].

By contrast, Tregs display a relative resistance to acidic stress, which is not merely passive but mechanistically grounded in their metabolic and signaling programs [45]. Tregs preferentially rely on OXPHOS and fatty acid oxidation, metabolic pathways that are less acutely sensitive to pH fluctuations than glycolysis [45]. Moreover, Tregs exhibit enhanced mitochondrial spare respiratory capacity, increased expression of antioxidant systems, and sustained activity of pH‐regulating transporters, allowing them to better preserve mitochondrial membrane potential and suppressive function under acidic conditions. Acidic environments may even reinforce Treg stability by promoting FOXP3 expression and limiting effector T cell–associated mTOR signaling, thereby conferring a selective advantage within the TME [45]. Memory T cells occupy an intermediate position along this pH‐sensitivity spectrum. Central and stem‐like memory subsets retain partial oxidative flexibility and mitochondrial fitness, which can buffer transient acidification [5]. However, chronic acidic stress has been reported to impair their long‐term persistence, recall responses, and epigenetic maintenance, suggesting that pH dysregulation may progressively erode durable antitumor immunity rather than acutely abolish function [5]. Notably, chimeric antigen receptor (CAR) T cells introduce an additional layer of complexity. CAR signaling often drives heightened tonic activation, sustained glycolytic flux, and elevated metabolic demand, rendering CAR‐T cells particularly vulnerable to acidic stress within solid tumors [46]. Preclinical studies indicate that extracellular acidosis and lactate accumulation can reduce CAR‐T cytotoxicity, persistence, and cytokine production, in part through impaired Ca^2+^ signaling and mitochondrial dysfunction [46]. Importantly, recent engineering strategies, including modulation of costimulatory domains, enhancement of mitochondrial biogenesis, or enforced expression of proton‐export or pH‐buffering mechanisms, have begun to improve CAR‐T resilience in acidic environments, highlighting pH as a modifiable barrier rather than an immutable limitation.

Collectively, these observations suggest that intracellular acidosis does not uniformly suppress T cells but instead reshapes the immune landscape by selectively disadvantaging highly glycolytic effector populations while favoring regulatory and metabolically adaptable subsets. This differential pH sensitivity contributes to a progressive skewing of immune composition and function at the IS, ultimately reinforcing tumor immune escape.

### 3.4. Intracellular Alkalinization in Tumor Cells: Actin Shielding and Resistance

On the tumor side of the IS, intracellular alkalinization promotes mechanical resistance. Cortical actin is reorganized into dense networks that physically block perforin pore formation [36]. Enhanced microtubule dynamics support trafficking of vesicles delivering immunosuppressive ligands, including PD‐L1 and galectins. Alkaline pH_i_ facilitates metabolic reprograming, increasing ATP availability to fuel actin turnover and resistance to cytotoxic stress [17, 22]. In multiple myeloma, proteasome inhibitor‐resistant clones display enhanced mitochondrial metabolism and altered pH regulation, contributing to therapy escape [47].

### 3.5. Bidirectional Dialog: A Hidden Checkpoint at the IS

When considered together, extracellular and intracellular pH form a hidden checkpoint at the IS (Figure 1). In T cells, intracellular acidosis leads to impaired Ca^2+^ signaling, defective cytoskeletal polarization, and reduced lytic granule release. In tumor cells, by contrast, intracellular alkalinization reinforces the actin shield, enhances metabolic robustness, and increases resistance to immune‐mediated killing. Beyond signaling, acidity regulates bidirectional metabolic exchanges at the IS. On the tumor to T cell side, extracellular acidosis restricts glucose availability for T cells, promoting nutrient competition; in addition, lactate accumulation suppresses T cell glycolysis and cytokine release [40]. Intercellular mitochondrial transfer, most commonly mediated by tunneling nanotubes (TNTs) or synaptic contacts, has been described in both physiological and pathological contexts [48, 49]. While no study to date has directly examined the role of extracellular or intracellular pH in regulating mitochondrial transfer, several lines of evidence strongly suggest pH‐sensitivity. Acidic extracellular pH disrupts actin dynamics, which are essential for TNT formation, and may thereby alter the frequency or stability of transfer events. Furthermore, acidosis imposes metabolic stress by impairing mitochondrial respiration and enhancing ROS production [50], conditions that are known triggers for mitochondrial uptake. At the IS, intracellular acidosis could inhibit SOCE [34], preventing optimal mitochondrial polarization [51, 52], which could increase susceptibility to mitochondrial drainage. Collectively, these observations support the hypothesis that both extracellular and intracellular pH may critically modulate mitochondrial transfer between T cells and tumor cells, a largely unexplored mechanism of immune dysfunction in acidic TMEs.

Figure 1Acidic pH as a hidden immune checkpoint: impact on the immunological synapse. (A) At physiological pH (pHe ~7.4), T cells form effective immunological synapses with cancer cells, characterized by actin polarization, TCR signaling, calcium influx, cytokine secretion, and lytic granule release, which leads to the killing of target cells. (B) Under acidic conditions (pH_e_ ~6.4–6.8), bidirectional changes in intracellular pH are observed: T cells tend to acidify, while cancer cells remain relatively alkalinized. These conditions may impair CRAC channel activity, TCR signaling, and cytoskeletal remodeling, thereby limiting effector functions. Cancer cells may simultaneously develop adaptive features that promote resistance. Additional mechanisms have been suggested, such as perforin inhibition, PD‐1/PD‐L1 interactions, or mitochondrial transfer via nanotubes, but remain to be fully confirmed. The schematic illustrations were designed using BioRender.com.

**Figure:**
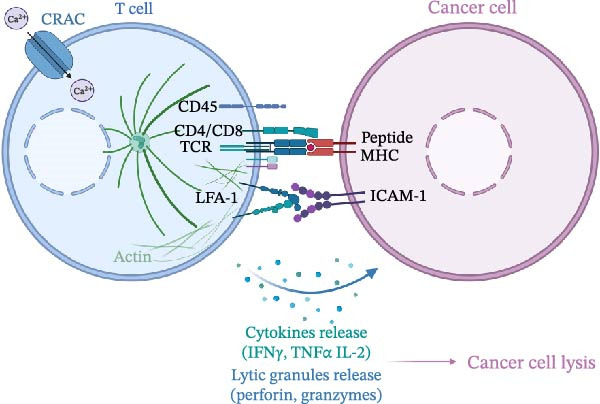
(A)

**Figure:**
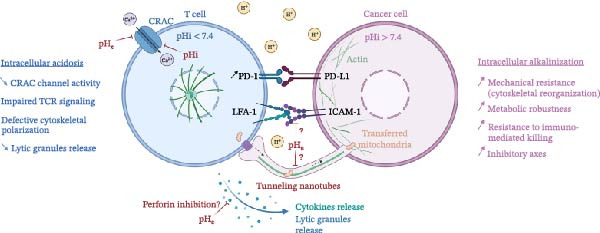
(B)

Taken together, acidic conditions promote a metabolic asymmetry in which T cells are functionally weakened, whereas tumor cells are metabolically reinforced. This dual mechanism would shift the outcome of IS formation toward tumor survival. Importantly, acidosis would not only act as a metabolic checkpoint but also increase the expression of classical immune checkpoints, including PD‐1 and CTLA‐4 on T cells and PD‐L1 on tumor cells, thereby amplifying immunosuppression [43, 53, 54]. This indicates that pH‐driven regulation intersects with, rather than acts independently of, canonical checkpoint pathways. Consequently, strategies aimed at buffering the TME or targeting pH‐sensitive signaling pathways may synergize with checkpoint blockade therapies to restore effective antitumor immunity.

### 3.6. Implications for Immunotherapy Resistance

Resistance to bispecific antibodies (e.g., CD3‐targeting) or CAR‐T therapy in cancer is commonly attributed to antigen loss, T cell exhaustion, or suppressive microenvironmental cues. We propose that pH dysregulation constitutes an additional, underappreciated mechanism of resistance. In acidic tumor niches, bispecific antibodies enforce prolonged T cell–tumor interactions, but acidosis metabolically could compromise T cells, potentially favoring mitochondrial transfer to cancer cells and accelerating exhaustion [55]. Similarly, CAR‐T cells, already vulnerable to exhaustion, may be further impaired by acidosis‐induced intracellular pH disturbances, reducing their persistence and cytotoxic potential. In parallel, regulatory T cells (Tregs), which are comparatively resistant to acidosis, may prevail in this context, thereby amplifying immunosuppression.

Acidic pH has also been shown to influence antigen expression and MHC class I stability in tumor cells, limiting antigen presentation and favoring immune escape [40, 55]. However, whether extracellular or intracellular acidosis directly drives antigen downregulation or alters the peptide, MHC landscape remains poorly understood [40]. Addressing how pH dynamics impact antigen density and MHC expression at the IS may reveal a previously overlooked determinant of resistance to bispecific antibodies and CAR‐T therapy.

### 3.7. pH Regulation Within the Broader Landscape of TME Constraints

While this review focuses on extracellular and pHi as a central regulator of IS function, pH remodeling does not occur in isolation within the TME. Instead, acidosis emerges as both a consequence and an amplifier of other well‐established immunosuppressive cues, including hypoxia, nutrient competition, ionic imbalance, and oxidative stress [34, 56, 57]. Hypoxia, a hallmark of poorly vascularized tumors, promotes a glycolytic shift in cancer cells through HIF‐1α–dependent transcriptional programs, leading to increased lactate production and proton extrusion via MCT transporters and carbonic anhydrases [40]. In this context, extracellular acidosis is not merely correlative but mechanistically downstream of hypoxic metabolism, while in turn reinforcing HIF‐1α stability and metabolic rewiring in both tumor and immune cells [40]. Metabolic competition for glucose further intersects with pH regulation at the IS. Glucose deprivation limits glycolytic flux in effector T cells, constraining ATP availability for actin remodeling [58], integrin activation, and sustained Ca^2+^ signaling [56, 59]. Acidosis could exacerbate this limitation by inhibiting key glycolytic enzymes and impairing mitochondrial function, thereby coupling nutrient scarcity to proton‐mediated suppression of synaptic stability. In parallel, lactate accumulation acts both as a metabolic substrate and a signaling molecule, directly inhibiting T cell proliferation and cytokine production while contributing to extracellular acidification [40]. Ionic dysregulation, particularly elevated extracellular potassium released from necrotic tumor cells, has also been shown to suppress TCR signaling and metabolic fitness [60]. Importantly, potassium accumulation and acidosis could converge on shared downstream targets, including membrane potential, Ca^2+^ influx through CRAC channels, and mitochondrial polarization, suggesting additive or synergistic effects at the IS [60–62]. Finally, ROS generated under hypoxic and metabolically stressed conditions interact bidirectionally with pH [63]. Acidic intracellular environments can impair antioxidant capacity, while mitochondrial dysfunction induced by low pHi enhances ROS production, further destabilizing cytoskeletal organization and signaling microclusters at the synapse [63].

Together, these observations position pH not as an isolated immunosuppressive factor but as an integrative checkpoint that coordinates metabolic, ionic, and mechanical constraints within the TME. Disentangling pH‐specific effects from cooccurring stressors, therefore, remains experimentally challenging, yet biologically relevant, as immune cells at the tumor interface are likely exposed to these signals simultaneously rather than sequentially.

### 3.8. Concluding Remarks

We define the “mechanical checkpoint” at the IS as a pH‐sensitive organizational layer that integrates adhesion, cytoskeletal dynamics, and force transmission to sustain productive T cell signaling (Figure 1). This platform operates at the subcellular scale of the synaptic interface and is composed of integrin‐ligand complexes (e.g., LFA‐1/ICAM‐1), the actin cytoskeleton and actomyosin contractility, membrane‐cytoskeleton linkers such as ERM proteins, and mechanoresponsive signaling hubs including CRAC (STIM1‐Orai1) channels. Small pH variations can disrupt this mechanically relevant platform by altering actin polymerization, integrin affinity, membrane tension, and Ca^2+^ influx, leading to unstable synapse architecture and impaired downstream signaling despite intact antigen recognition.

Despite compelling evidence, major gaps remain. No direct studies have monitored pH_i_ in T cells during IS formation under acidic pH_e_. The coupling between tumor cell intracellular alkalinization and actin shielding at the IS remains hypothetical. The interplay between bispecific antibody, stabilized synapses, and acidosis‐driven mitochondrial transfer has not been experimentally addressed. Microsystems capable of real‐time pH monitoring would offer a unique opportunity to fill these gaps to directly assess pH‐driven resistance mechanisms.

Extracellular acidosis and intracellular alkalinization form a bidirectional checkpoint at the IS, weakening T cells while reinforcing tumor defenses. This metabolic asymmetry contributes to resistance against bispecific antibodies and CAR‐T therapy, especially in the acidic TME. Addressing this hidden checkpoint requires innovative approaches, including microsystems models, pH‐sensitive imaging, and combinatorial therapies targeting both immune checkpoints and pH regulation.

## 4. Methodological Approaches to Study Intra‐ and Extracellular pH During IS Formation

### 4.1. Measuring Extracellular pH at the IS

The acidic TME generates strong extracellular pH gradients, which may reach values as low as 6.4 within confined niches [39]. Standard techniques for measuring pH in bulk samples include pH electrodes and microelectrodes; however, these approaches lack the spatial resolution necessary to capture the nanoscale pH changes at the IS. Fluorescent pH indicators, such as SNARF‐1 or BCECF, enable real‐time monitoring of pH with ratiometric accuracy [64]. These dyes can be conjugated to dextrans or lipids to restrict them to the extracellular compartment [65, 66]. pH‐sensitive nanoparticles have been developed to map extracellular acidification at single‐cell resolution, offering improved stability over traditional dyes [13, 23, 67]. However, none of these tools has yet been applied directly to the immune synapse. Capturing the acidic microdomains formed under the T cell membrane during contact with tumor cells remains a methodological challenge [68].

### 4.2. Imaging Intracellular pH in T Cells and Tumor Cells

The monitoring of intracellular pH is crucial to understanding how extracellular acidosis translates into functional changes within immune and tumor cells. Ratiometric fluorescent probes such as BCECF‐AM or SNARF‐AM remain the gold standard for single‐cell pHi measurements [64]. Genetically encoded pH sensors, such as pHluorin (a GFP variant), enable long‐term monitoring of intracellular pH in defined cell types [69]. These tools can be targeted to specific organelles, such as endosomes or mitochondria, to dissect compartment‐specific acid–base dynamics. Noninvasive magnetic resonance spectroscopic (MRS) provides a label‐free, quantitative assessment of pH_i_ [70–72], though its resolution is insufficient for IS. In T cells, dynamic pH_i_ monitoring during IS formation has not been systematically performed. Such approaches could reveal whether intracellular acidification precedes calcium signaling defects or exhaustion onset.

### 4.3. Simultaneous Measurement of pH and Calcium Fluxes

Given the intimate coupling between pH regulation and calcium signaling, combined readouts are essential. Dual‐imaging strategies using pHluorin (pH) and GCaMP (Ca^2+^) [73] or Fura‐2 (Ca^2+^) can reveal real‐time interactions between proton fluxes and Ca^2+^ signals [74]. Confocal and two‐photon microscopy allow high‐resolution imaging of pH_i_ and Ca^2+^ within 3D tissue models, including lymph node slices [74, 75]. Yet, to date, no study has visualized pH and calcium simultaneously at the IS in the context of tumor immunity, a clear methodological gap.

### 4.4. Microsystems for Potential pH Studies at the IS

Microfluidics offers unparalleled opportunities to precisely control extracellular acidity, especially with patient samples [76]. Microfluidic systems can trap T cell–tumor pairs under possibly defined pH conditions, enabling high‐throughput single‐cell readouts [76]. Controlled perfusions could create stable pH conditions across microchannels, mimicking acidic tumor niches. Such systems provide the possibility to dissect how extracellular acidosis alters IS formation, Ca^2+^ oscillations, and mitochondrial transfer in real time, under conditions that approximate the TME niche.

### 4.5. Nanosensors and Novel Technologies

The emergence of nanotechnological sensors adds new possibilities for probing pH at unprecedented resolution [77]. Quantum dot‐based sensors exhibit high photostability and can be engineered to report pH fluctuations at the nanometer scale [77]. FRET‐based nanosensors targeted to specific cell membranes have been used to visualize proton fluxes at contact sites between cells [78]. Their application to T cell–tumor synapses could illuminate localized pH dynamics during immune attack. Atomic force microscopy (AFM) could be used with pH‐sensitive probes to map local proton concentrations at the cell surface [79]. Integrating AFM with IS studies could couple pH gradients with mechanical force measurements.

### 4.6. Challenges and Limitations

Despite these significant advances, important methodological limitations must be considered when interrogating pH dynamics at the IS. Classical ratiometric fluorescent dyes such as SNARF‐1 or BCECF remain widely used but are prone to intracellular leakage, uneven compartmentalization, and calibration variability across cell types and experimental conditions [64]. These limitations complicate quantitative comparisons, particularly in highly dynamic settings such as synapse formation, where rapid pH fluctuations may occur on subsecond timescales. Genetically encoded pH reporters, including pHluorin‐based sensors, offer improved cellular specificity and long‐term imaging capacity but are constrained by their intrinsic pKa and limited dynamic range in acidic environments [69]. As a result, pH changes within the lower range typical of the TME may be underestimated, especially in chronically acidified or exhausted T cells. Moreover, the availability of T cell subset–specific or activation state–restricted pH reporters remains limited, restricting precise mapping of intracellular acidification during differentiation or dysfunction. Spatial resolution poses an additional challenge. Most current approaches report bulk extracellular or intracellular pH, lacking the spatial precision required to resolve the nanometer‐scale synaptic cleft, which is estimated to be only ~15–100 nm wide. Consequently, local proton gradients at the core of the IS are likely masked by averaging effects, underscoring the need for synapse‐targeted or membrane‐anchored pH probes. Microfluidic and IS‐on‐chip platforms have enabled the controlled manipulation of pH and metabolites; yet, these systems are not without caveats. Shear stress and mechanical confinement can alter cytoskeletal dynamics, integrin engagement, and synapse stability, particularly in primary human T cells, potentially confounding the interpretation of pH‐specific effects. Careful benchmarking against static or low‐shear conditions is therefore essential. Nanosensor‐based and nanobody‐conjugated approaches offer promising routes to probe local pH with high spatial resolution but face practical limitations in highly scattering tumor tissues, where signal‐to‐noise ratios can be severely reduced. In addition, nanoparticle uptake, surface charge, and functionalization may themselves influence membrane dynamics or cellular metabolism, necessitating rigorous controls. Finally, although emerging studies have begun to explore mitochondrial transfer and metabolic coupling at the IS, no published work has yet achieved simultaneous real‐time monitoring of extracellular pH, intracellular pH, and intercellular mitochondrial dynamics during synapse formation. Bridging this gap will require multimodal imaging strategies and careful integration of pH, Ca^2+^, and metabolic readouts.

Collectively, these limitations do not diminish the relevance of pH as a regulatory signal but rather highlight the technical challenges inherent to dissecting proton dynamics at the immune synapse. Explicit acknowledgment of these constraints is crucial for experimental design, data interpretation, and the development of next‐generation tools that can resolve pH‐dependent immune regulation with higher spatial and temporal precision.

### 4.7. Perspectives: Methodological Innovations for IS Studies

Advancing the study of the IS requires the integration of multiparametric readouts at single‐cell resolution, including real‐time monitoring of extracellular and intracellular pH, Ca^2+^ fluxes, cytoskeletal remodeling, and lytic granule polarization and release. Selective microfluidic and IS‐on‐chip platforms will be instrumental in delivering drugs, metabolites, and cytokines under precisely defined extracellular acidity, enabling controlled dissection of cell type‐specific and subset‐specific responses. When applied to patient‐derived immune and tumor cells, these approaches may allow direct correlation between pH‐driven IS dysfunction and clinical resistance to bispecific antibodies or CAR‐T cell therapies. Importantly, future IS studies must move beyond reductionist two‐dimensional systems toward more complex and programmable models that better recapitulate the immunosuppressive tumor niche.

Artificial biomimetic synapse platforms [80], including supported lipid bilayers [81], tunable polymeric substrates, and emerging graphene oxide‐based interfaces [80, 82], offer a unique opportunity to control antigen density, ligand mobility, mechanical stiffness, and metabolic priming in a modular fashion. The programmable nature of these systems makes them particularly well suited to integrating pH as a tunable variable, for example, by imposing localized extracellular acidosis or dynamic pH gradients at the synaptic interface. Such platforms could disentangle pH‐specific effects from confounding microenvironmental cues and reveal how regulatory T cells maintain suppressive function under acidic stress, while effector T cells progressively lose synaptic stability and cytotoxic competence.

Methodological advances in pH imaging, genetically encoded reporters, and micro‐ and nanoengineered platforms now provide unprecedented opportunities to interrogate the “hidden checkpoint” imposed by acidity at the IS. Nevertheless, the direct and simultaneous integration of intra‐ and extracellular pH monitoring with IS dynamics, Ca^2+^ signaling, and intercellular metabolic exchange remains largely uncharted territory. Overcoming current technical limitations, such as probe dynamic range, spatial resolution within the synaptic cleft, and mechanical perturbations introduced by microsystems, will be essential to fully capture proton dynamics at the nanoscale.

By combining pH‐sensitive sensors, calcium imaging, cytoskeletal reporters, and programmable microsystems, future studies could unravel how extracellular acidosis and tumor‐intrinsic intracellular alkalinization reshape the mechanical and metabolic dialog between T cells and cancer cells at the IS. Such integrative approaches would pave the way for therapeutic strategies aimed at normalizing or exploiting pH gradients to restore T cell function, enhance synaptic efficacy, and ultimately overcome resistance to cancer immunotherapies.

## 5. Therapeutic Approaches to Overcome pH‐Driven Immunosuppression

While a wide range of strategies has been proposed to counteract pH‐driven immunosuppression, these approaches differ markedly in their level of experimental validation, clinical readiness, and systemic feasibility. Some interventions are already clinically approved for other indications and could be rapidly repurposed, whereas others remain at an early preclinical or conceptual stage. Moreover, targeting pH regulation raises important concerns regarding off‐target toxicity and tumor adaptive responses. The main therapeutic strategies discussed below are summarized and ranked by mechanism of action, advantages, limitations, and current stage of development (Table 1).

**Table 1:** Therapeutic strategies targeting pH‐driven immunosuppression at the immunological synapse.

Strategy	Target/mechanism	Advantages	Major limitations and toxicities	Tumor adaptation	Developmental status
Systemic or local buffering (bicarbonate and Tris‐base) [23, 72, 83]	Neutralization of extracellular acidity	Simple, inexpensive; improves CD8^+^ infiltration; synergy with ICIs	Poor tumor specificity; risk of metabolic alkalosis	Increased glycolysis; upregulation of proton exporters	Preclinical/early translational
Proton pump inhibitors (PPIs) [84, 85]	Inhibition of V‐ATPases	Clinically approved drugs; reduce acidosis and drug resistance	Broad systemic effects; variable bioavailability	Compensatory transporter expression	Preclinical/repurposing stage
NHE1 inhibition [86]	Blockade of proton extrusion and actin remodeling	Reduces invasion and cytoskeletal dynamics	High systemic toxicity; essential in normal tissues	Alternative pH regulators activated	Preclinical
MCT1/4 inhibition [42, 87, 88]	Block lactate/proton export	Restores T cell function; targets tumor metabolism	Muscle, cardiac toxicity; metabolic inflexibility	Switch to alternative fuels	Preclinical/early clinical
CAIX inhibition [89, 90]	Hypoxia‐induced pH regulation	Tumor‐selective expression; synergy with PD‐1 blockade	Hypoxia heterogeneity; adaptive enzyme redundancy	Upregulation of other carbonic anhydrases	Preclinical/early clinical
Metabolic modulators (metformin and LDH inhibitors) [19, 91]	Shift glycolytic flux and lactate production	Clinically accessible (metformin); immune‐supportive	Systemic metabolic effects; modest potency	Metabolic rewiring	Preclinical/repurposing
pH‐responsive nanomedicine [92–95]	Drug release in acidic niches	High tumor selectivity; reduced systemic toxicity	Manufacturing complexity; biodistribution challenges	Heterogeneous pH landscapes	Preclinical
Engineering pH‐resistant T cells [41, 96, 97]	Enhance metabolic and pH resilience	Directly addresses immune dysfunction	Manufacturing complexity; regulatory hurdles	Tumor counteradaptation	Preclinical/early translational

### 5.1. Buffering the Acidic TME

One of the earliest strategies to counteract extracellular acidity has been the use of systemic or local buffers. Oral or intravenous administration of sodium bicarbonate was shown to increase tumor pH_e_ and reduce metastasis in preclinical models [72]. Similarly, Tris‐base supplementation improved intratumoral pH and synergized with checkpoint inhibitors in multiple cancer models [83]. However, systemic alkalinization raises safety concerns, such as metabolic alkalosis, and lacks specificity for the tumor site [72]. Another approach involves the use of proton pump inhibitors (PPIs), such as omeprazole and lansoprazole, which inhibit V‐ATPase. These compounds neutralize the extracellular space, reduce drug resistance, and favor T cell infiltration [84, 98]. Yet, their broad effects and pharmacokinetic variability limit clinical translation.

### 5.2. Targeting pH Regulators in Tumor and Immune Cells

The maintenance of intracellular alkalinity and extracellular acidosis in tumors is largely sustained by pH‐regulating transporters, making them attractive therapeutic targets [99]. NHE1 is frequently overexpressed in cancers, where it drives proton efflux and extracellular acidification [100]. Its inhibition disrupts actin cytoskeleton remodeling and reduces tumor cell invasion [101]. V‐ATPases, beyond their role in buffering, are essential for lysosomal acidification; selective inhibition induces tumor cell apoptosis and enhances antigen presentation [85]. MCT1‐4 sustain glycolysis and lactate export, fueling tumor acidosis [40]. Targeting MCT has been shown to restore T cell function under acidic conditions and sensitize tumors to immune attack [19, 40, 87]. CAIX, a hypoxia‐inducible enzyme, maintains acidic extracellular pH, and its inhibition enhances T cell infiltration while synergizing with PD‐1 blockade in preclinical models [89]. Despite these promising findings, the therapeutic exploitation of pH regulators must address a major challenge: achieving tumor selectivity while minimizing systemic toxicity, since these transporters are also critical for normal tissue homeostasis.

### 5.3. Modulating Metabolism to Reprogram pH

Extracellular acidity largely stems from tumor glycolysis and lactate export. Strategies to redirect metabolism may therefore correct pH and improve immunity. Glycolysis inhibitors such as 2‐deoxyglucose reduce proton production but face toxicity concerns in highly glycolytic normal tissues [102]. Lactate dehydrogenase (LDH) inhibitors lower lactate production, decreasing both acidosis and immunosuppression [103]. Metformin, by targeting mitochondrial complex I, shifts metabolism away from aerobic glycolysis and has been reported to normalize TME pH while enhancing CD8^+^ T cell responses [91]. Of note, tumor–T cell competition for glucose exacerbates acidosis and immune dysfunction [56]. Correcting metabolic imbalances may thus indirectly normalize IS function.

### 5.4. Nanomedicine and pH‐Responsive Drug Delivery

The acidic TME can be exploited to design pH‐responsive nanocarriers that release drugs specifically at low pH, minimizing systemic toxicity. pH‐sensitive liposomes release their content in acidic niches, improving tumor drug delivery [92]. Polymeric nanoparticles with protonatable groups exhibit charge‐switching properties, enhancing penetration into acidic tumors [93, 94, 104]. pH‐activatable immunotherapy carriers have been developed to release checkpoint inhibitors only in acidic microdomains, thereby enhancing efficacy and limiting systemic autoimmunity [95]. Such strategies could be adapted to selectively deliver buffering agents or metabolic inhibitors at the immune synapse, opening a novel therapeutic dimension.

### 5.5. Integrating pH Modulation With Immunotherapy

Several studies suggest that correcting tumor acidosis may synergize with checkpoint blockade and adoptive T cell therapy [83]. In melanoma, systemic buffering improved anticancer efficacy by enhancing CD8^+^ infiltration [105]. CAR‐T cells exposed to acidic pH display impaired cytotoxicity, reduced granule release, and decreased persistence [96]. One promising avenue is the use of cellular and molecular engineering to generate T cells resistant to acidosis, for example, by modulating pH regulators [97, 99], or by reprogramming metabolic pathways to sustain effector function despite extracellular acidity [45]. Another strategy involves engineering “armored” CAR‐T cells to secrete cytokines (e.g., IL‐2 or IL‐15) that are designed to maintain functional activity in acidic environments [41]. Such pH‐protected cytokine delivery may help mitigate acidosis‐driven T cell dysfunction and support effector persistence. Bispecific antibodies, which enforce stable synaptic contacts, could paradoxically favor mitochondrial transfer under acidic stress, accelerating T cell exhaustion, a hypothesis that remains to be experimentally tested. Although BCMA × CD3 bispecific antibodies show robust clinical activity in multiple myeloma despite the acidic bone marrow niche [47, 106], this does not imply that pH‐driven IS dysfunction is irrelevant in hematologic settings. Rather, current evidence suggests that the impact of acidosis may be attenuated or partially compensated in this context. T cells within the bone marrow may display increased tolerance to chronic metabolic stress, while the absence of dense extracellular matrix constraints could reduce the mechanical sensitivity of hematologic synapses to pH‐dependent cytoskeletal perturbations. Whether extracellular acidity contributes to primary or acquired resistance to bispecific antibodies remains unclear and warrants direct investigation. These considerations highlight the context‐dependent nature of pH‐mediated IS regulation and caution against extrapolating findings from solid tumors to hematologic malignancies. Together, these findings argue for combined approaches that integrate immunotherapy with pH modulation or cellular engineering to bypass this hidden immune checkpoint imposed by acidity.

### 5.6. Perspectives: Toward Precision pH‐Immunotherapy

Despite significant advances, several critical questions remain. What are the precise spatiotemporal dynamics of pH_e_ and pH_i_ at the IS? How does pH modulate bidirectional cytoskeletal remodeling and mitochondrial trafficking? Can pH‐correcting agents be optimized for selective delivery to immune synapses? We propose that microfluidic single‐synapse systems, capable of selective drug delivery to T cells or tumor cells, combined with real‐time pH and Ca^2+^ imaging, provide an ideal platform to answer these questions. By integrating patient‐derived samples (diagnosis vs. relapse and immunotherapy resistance), such approaches could reveal predictive signatures of pH‐driven dysfunction and guide rational combination therapies.

In conclusion, extracellular acidity represents an underappreciated barrier to effective antitumor immunity, functioning as a metabolic checkpoint that destabilizes the IS. Targeting pH regulation through buffers, transporter inhibitors, metabolic reprogramming, and pH‐responsive nanomedicine holds strong potential to restore T cell function and synergize with immunotherapies. However, tumor adaptation to pH‐correcting strategies, through increased glycolytic flux, transporter redundancy, or metabolic rewiring, represents a major obstacle that will likely necessitate combination therapies rather than single‐agent approaches (Table 1). In addition, methodological gaps remain, particularly the absence of tools to monitor intra‐ and extracellular pH in real time at the IS. Addressing this limitation through microsystems and imaging innovations could open new therapeutic avenues and translate into more durable responses for cancer patients.

## 6. Conclusions/Perspectives

The acidic TME likely establishes a bidirectional, pH‐dependent checkpoint at the IS. Converging, but still fragmentary, evidence suggests that low extracellular pH can weaken T cells by reducing LFA‐1/ICAM‐1‐dependent adhesion, delaying actin clearance at the c‐SMAC, and inhibiting store‐operated Ca^2+^ entry, thereby curtailing cytokine production and lytic granule delivery. In parallel, many tumor cells maintain an alkaline intracellular pH via proton and lactate exporters (e.g., NHE1, MCT1/4, and CAIX), which reinforces a dense cortical actin “shield,” optimizes bioenergetics, and resists perforin‐mediated damage. While this asymmetric tuning, acidic outside, alkaline inside, offers a coherent working model that can account for resistance to checkpoint inhibitors, bispecific antibodies, and CAR‐T therapies, direct, spatiotemporally resolved demonstrations at bona fide tumor cell–T cell synapses remain limited. Taken together, current evidence supports a conceptual, testable hierarchy in which pH fluctuations may act as an upstream perturbation at IS. In this model, extracellular and intracellular acidification could first attenuate store‐operated Ca^2+^ entry through CRAC channels, thereby limiting mitochondrial Ca^2+^ uptake and compromising mitochondrial polarization and metabolic output. Reduced ATP availability and increased oxidative stress would then be expected to impair actin remodeling, integrin activation, and force transmission at the synapse. Such a sequence would ultimately destabilize synapse architecture and limit centrosome polarization and lytic granule release. Importantly, mitochondrial dysfunction in this framework is proposed not as a sole initiating event but as a downstream amplifier that reinforces mechanical failure through a feed‐forward loop. Although this stepwise cascade remains conceptual, it provides a coherent framework to integrate metabolic and mechanical defects observed under acidic conditions and highlights specific nodes, Ca^2+^ influx, mitochondrial fitness, and cytoskeletal dynamics that can be experimentally interrogated in future studies (Figure 2).

Figure 2Hypothetical stepwise pH‐driven hierarchy at the immunological synapse. Proposed conceptual model illustrating how extracellular and intracellular pH fluctuations may sequentially impair immunological synapse (IS) function in T cells (A) and cancer cells (B). In T cells (A), acidification is hypothesized to first attenuate store‐operated Ca^2+^ entry through CRAC (STIM1‐Orai1) channels, leading to reduced mitochondrial Ca^2+^ uptake, mitochondrial depolarization, and diminished metabolic output. These metabolic alterations are proposed to weaken actin remodeling, integrin activation, and force transmission at the synapse, failing the mechanical checkpoint, defective centrosome polarization, and impaired lytic granule exocytosis. In cancer cells (B), extracellular acidosis combined with tumor‐intrinsic intracellular alkalinization is proposed to reshape synapse‐associated processes through largely hypothetical mechanisms. Acidic extracellular pH may modulate plasma membrane signaling and Ca^2+^ handling, while intracellular alkalinization could favor cytoskeletal reinforcement via actin remodeling pathways, contributing to the formation of a protective cortical “actin shield.” In parallel, extracellular acidosis has been shown in multiple tumor models to promote immune evasion by increasing the expression of immune checkpoint ligands such as PD‐L1, decreasing antigen presentation, whereas a direct contribution of intracellular alkalinization to checkpoint regulation remains speculative. Tumor‐side pH remodeling may also influence mitochondrial positioning, metabolic resilience, and potentially intercellular mitochondrial transfer at the synapse, although these processes remain incompletely defined. Solid arrows indicate established interactions, whereas dashed arrows denote proposed or incompletely validated links. The schematic illustrations were designed using BioRender.com.

**Figure:**
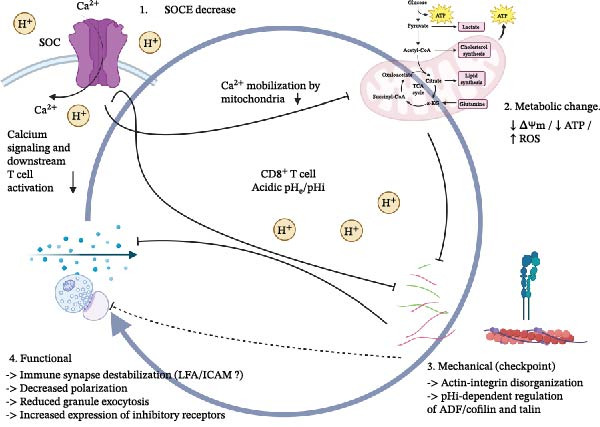
(A)

**Figure:**
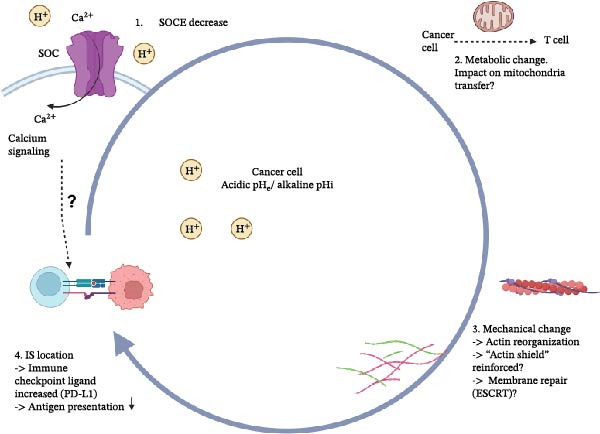
(B)

**Figure 3:**
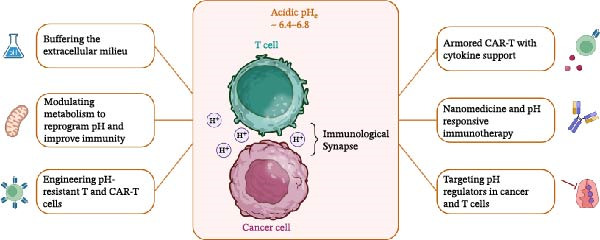
Therapeutic strategies to overcome pH‐driven immune synapse dysfunction. Schematic representation of potential interventions designed to restore T cell effector function within an acidic tumor microenvironment (pH_e_ ~ 6.4–6.8). Approaches include buffering strategies to normalize extracellular pH, metabolic reprogramming of tumor and immune cells, and engineering of T cells (including CAR‐T cells) with enhanced resistance to acidosis or endowed with additional effector functions. Nanoparticle‐based delivery systems responsive to acidic pH are also under investigation to achieve selective drug release at the synapse. Other strategies aim to combine pH modulation with immune checkpoint blockade or with bispecific antibodies to improve T cell activation. Together, these approaches seek to reprogram the immunological synapse and mitigate the inhibitory effects of tumor acidity on antitumor immunity. The schematic illustrations were designed using BioRender.com.

In this review, we delineate the key experimental gaps, real‐time mapping of extracellular and intracellular pH within the synaptic cleft, its coupling to Ca^2+^ flux, mitochondrial positioning/transfer, and cytoskeletal remodeling across T cell subsets, and outline enabling platforms and therapeutic strategies (Figure 3) to test and refine this model. Therapeutically, reframing acidosis as a druggable checkpoint opens multiple routes: buffering strategies; targeted inhibition of NHE1, MCT1/4, V‐ATPase, or CAIX; metabolic rewiring to limit proton production; pH‐responsive drug delivery; and engineering of pH‐resilient effector cells. Rational combinations that normalize pH while blocking canonical checkpoints should restore synapse stability, reinvigorate cytotoxic function, and improve durability of response. Integrating quantitative pH biomarkers into trial design will be critical to translating these concepts into precision immuno‐oncology.

## Author Contributions

Conceptualization: Yasmine Touil. Writing – original draft preparation: Yasmine Touil. Writing – review and editing: Aurélie Guillemette, Eva Gez, Sofia Titah, and Yasmine Touil. Visualization: Aurélie Guillemette, Eva Gez, and Sofia Titah. Supervision: Yasmine Touil. Project administration: Yasmine Touil. Funding acquisition: Yasmine Touil.

## Funding

This research was funded by grants from INCA_18477, INSERM, Ligue contre le cancer, and the University of Lille. Sofia Titah is financed by INSERM and by the Hauts‐de‐France Region. Eva Gez is financed by INCA.

## Disclosure

All authors have read and agreed to the published version of the manuscript.

## Conflicts of Interest

The authors declare no conflicts of interest.

## Data Availability

Data sharing is not applicable to this article as no datasets were generated or analyzed during the current study.
